# Efficacy of five-step shoulder manipulation for rotator cuff-related shoulder pain: protocol for a multicenter randomized controlled trial

**DOI:** 10.1186/s13063-023-07540-5

**Published:** 2023-08-07

**Authors:** Shuang Liu, Jin-Tao Liu, Lin Chen, Tian-You Fan, Xue-Jun Cui, Shao-Dan Cheng, Yan-Jiao Chen, Qi Shi, Chun-Chun Xue, Xiao-Feng Li

**Affiliations:** 1 Shanghai Municipal Hospital of Traditional Chinese Medicine, Shanghai University of Traditional Chinese Medicine, 274 Zhijiangzhong Road, Shanghai, 200071 China; 2 LongHua Hospital Shanghai University of Traditional Chinese Medicine, 725 Wanpingnan Road, Shanghai, 200032 China; 3 Suzhou Hospital of Traditional Chinese Medicine, 899 Wuzhongxi Road, Suzhou, 215009 Jiangsu China; 4 Shanghai GuangHua Hospital of Integrated Traditional Chinese and Western Medicine, 540 Xinhua Road, Shanghai, 200052 China; 5 Shanghai Research Institute of Acupuncture and Meridian, YueYang Hospital of Integrated Traditional Chinese and Western Medicine, 650 Wanpingnan Road, Shanghai, 200030 China; 6 Qi Shi’s Studio of Famous Chinese Medicine Physician, 274 Zhijiangzhong Road, Shanghai, 200032 China

**Keywords:** Manipulation, Rotator cuff-related shoulder pain, Five-step shoulder manipulation, Exercises, Randomized controlled trials

## Abstract

**Background:**

Rotator cuff-related shoulder pain (RCRSP) is the most common cause of shoulder disorders. In China, manipulation has been used extensively for the treatment of patients with RCRSP. However, high-quality clinical evidence to support the therapeutic effect of manipulation is still limited.

**Methods:**

A multicenter, participant-, outcome assessor-, and data analyst-blinded, randomized, placebo-controlled trial will be conducted. A total of 280 participants with RCRSP will be recruited from three hospitals and randomly assigned to a five-step shoulder manipulation (FSM) group or a sham manipulation (SM) group. Each group will receive four weekly treatment sessions, with all participants performing exercises at home for 12 weeks. Assessments, namely the Constant–Murley score, visual analog scale, range of motion, and 36-Item Short Form Survey, will be made at baseline, 4, 12, 18, and 24 weeks. Adverse events during the study will also be recorded.

**Discussion:**

This is a pragmatic clinical trial to evaluate the efficacy and safety of FSM in patients with RCRSP. The findings of this study will provide worthy clinical evidence for manual therapy for RCRSP.

**Trial registration:**

China Registered Clinical Trial Registration Center ChiCTR2000037577. Registered on 29 August 2020.

**Supplementary Information:**

The online version contains supplementary material available at https://doi.org/10.1186/s13063-023-07540-5.

## Administrative information

**Table:** 

Title {1}	Efficacy of five-step shoulder manipulation for rotator cuff-related shoulder pain: protocol for a multicenter randomized controlled trial
Trial registration {2a and 2b}	China Registered Clinical Trial Registration Center ChiCTR2000037577. Registered on 29 August 2020 and updated on July 18 2022. Details of the trial registration are shown in Supplementary Table 1
Protocol version {3}	Protocol version 2.0, June 19, 2022
Funding {4}	Medical Innovation Project of Shanghai Science and Technology Commission (No. 21Y11921300). Domestic Science and Technology Cooperation Project of Shanghai Science and Technology Innovation Action Plan (No. 22015830700). Shanghai University of TCM Excellent Talents Training Program (TCM[2020]10) and Xinglin Young Talent Training System-Xinglin Scholars Project (TCM[2020]23). Future plans of Shanghai Municipal Hospital of TCM (No. WLJH2021ZY-ZLZX001, GZS001, MZY034)
Author details {5a}	Shuang Liu: Department of Orthopedics, Shanghai Municipal Hospital of Traditional Chinese Medicine, Shanghai University of Traditional Chinese Medicine; Institute of Spinal Disease, LongHua Hospital Affiliated to Shanghai University of Traditional Chinese Medicine Jintao Liu: Department of Orthopaedics, Suzhou Hospital of Traditional Chinese Medicine Lin Chen: Department of Orthopedics, Shanghai Municipal Hospital of Traditional Chinese Medicine, Shanghai University of Traditional Chinese Medicine Tianyou Fan: Department of Orthopedics, Shanghai Municipal Hospital of Traditional Chinese Medicine, Shanghai University of Traditional Chinese Medicine Xuejun Cui: Institute of Spinal Disease, LongHua Hospital Affiliated to Shanghai University of Traditional Chinese Medicine Shaodan Cheng: Department of Rehabilitation, Shanghai GuangHua Hospital of Integrated Traditional Chinese and Western Medicine Yanjiao Chen: Shanghai Research Institute of Acupuncture and Meridian, YueYang Hospital of Integrated Traditional Chinese and Western Medicine Qi Shi: Institute of Spinal Disease, LongHua Hospital Affiliated to Shanghai University of Traditional Chinese Medicine; Qi Shi’s Studio of Famous Chinese Medicine Physician Chunchun Xue: Department of Pain, Shanghai Municipal Hospital of Traditional Chinese Medicine, Shanghai University of Traditional Chinese Medicine Xiaofeng Li: Department of Orthopedics, Shanghai Municipal Hospital of Traditional Chinese Medicine,Shanghai University of Traditional Chinese Medicine; Qi Shi’s Studio of Famous Chinese Medicine Physician
Name and contact information for the trial sponsor {5b}	Xiaofeng Li, Shanghai Municipal Hospital of Traditional Chinese Medicine, Shanghai University of Traditional Chinese Medicine, Shanghai 200071, China. E-mail: lixiaofeng0409@163.com
Role of sponsor {5c}	The study sponsor will designate relevant researchers to conduct the study, collect, manage and analyze the data, and publish the results in a peer-reviewed journal. Funders do not participate in this study

## Introduction

### Background and rationale {6a}

Shoulder pain is the third most common type of musculoskeletal disorder, with a point prevalence of up to 26% [1]. Rotator cuff-related shoulder pain (RCRSP) is an all-encompassing term that comprises a spectrum of shoulder conditions, such as subacromial impingement syndrome, rotator cuff tendinopathy, and symptomatic rotator cuff tears [2–4], contributing to over two thirds of the occurrences of shoulder pain [5, 6]. Pain and loss of motion and strength in the shoulder are the most common complaints of RCRSP patients [7], limiting their capacity for self-care and employment [8]. The guidelines recommend conservative management prior to injection or seeking a surgical opinion [7, 9, 10].

Manipulation, as a classical type of conservative intervention, achieves a therapeutic effect by aiming to create balance in the muscles and bones of the human body. Generally, a Chinese medicine practitioner uses their hands or different parts of the body to manipulate a specific part of the patient’s body. In China, manipulation has been used widely for patients with RCRSP. The symptoms of RCRSP are believed to result from an imbalance between the muscles and bones caused by tendon malposition and joint subluxation (International Classification of Diseases (ICD) 11th Revision, code: ICD-11). To achieve balance, we devised a five-step shoulder manipulation (FSM) based on the “muscle and bone balance” theory of traditional Chinese medicine (TCM). Our previous study showed that FSM was beneficial in alleviating pain and improving the function of the shoulder in RCRSP patients [11]; however, the study lacked high-quality clinical evidence, had a limited sample size, and was not randomized. To evaluate the efficacy and safety of FSM for RCRSP, we designed this randomized controlled trial (RCT) to focus on pain and functional recovery in the shoulder, providing clinical support for choosing interventions in real-world clinical practice.

#### Objectives {7}

The objective of this study is to evaluate the efficacy and safety of FSM in the treatment of RCRSP.

#### Trial design {8}

This study is a two-arm parallel group, multicenter, participant-, outcome assessor-, and data analyst-blinded, randomized, placebo-controlled trial with a superiority, which includes outcome assessments at 5 time points (baseline, 4, 12, 18, and 24 weeks) over a 24-week period. All eligible patients will be enrolled in the baseline evaluation and then randomly assigned into two groups on a 1:1 basis. One group will be treated with FSM, and the other with sham manipulation (SM). In parallel, patients in both groups will be required to perform home-based exercise as the basic treatment. During the study, Constant-Murley score (CMS), visual analog scale (VAS), full shoulder range of motion (ROM), and 36-Item Short Form Survey (SF-36) will be evaluated. The independent outcome assessors will be responsible for assessing the outcomes.

## Methods: participants, interventions, and outcomes

### Study setting {9}

A total of 280 individuals with RCRSP will be recruited from three hospitals in China: (1) Shanghai Municipal Hospital of TCM, (2) LongHua Hospital Affiliated to Shanghai University of TCM, and (3) Shanghai GuangHua Hospital of Integrated TCM and Western Medicine. These three hospitals are grade III, class A, teaching hospitals affiliated to Shanghai University of TCM, and they are all located in the center of Shanghai, with convenient transportation, good medical conditions, and plentiful patient resources. These conditions will be conducive to the smooth development of the study.

#### Eligibility criteria {10}

*Inclusion criteria*

- ✧ Age 40–70 years old;
- ✧ Pain in the anterolateral acromial area or pain with active shoulder elevation [12], with symptoms lasting for more than 3 months;
- ✧ Positive signs in each of the following 4 categories: pain when resisting humeral external rotation or abduction, Jobe test, Hawkins test, Neer test;
- ✧ VAS score ≥ 40 mm.

*Exclusion criteria*

- ✧ Clinical signs of massive rotator cuff tears (a tear greater than 5 cm in diameter) or the presence of two or more tendon tears [13, 14];
- ✧ Glenohumeral osteoarthritis (OA) or other inflammatory arthritis;
- ✧ Frozen shoulder;
- ✧ Fracture or dislocation of shoulder;
- ✧ Previous neck or shoulder surgery;
- ✧ Osteoporosis;
- ✧ Neurological disorders;
- ✧ Tumor;
- ✧ Pregnancy.

#### Who will take informed consent? {26a}

Before the screening, potentially eligible patients will be informed in detail of the purpose, procedure, potential risks, and benefits of the trial. Eligible patients will have 1 week to fully consider whether to participate in the study and will need to sign two informed consents, one held by the researcher and the other by the patient, if they wish to participate.

#### Additional consent provisions for collection and use of participant data and biological specimens {26b}

N/A. No biological specimens will be involved in this study.

## Interventions

### Explanation for the choice of comparators {6b}

The SM is the control treatment in this study and is a reliable placebo that achieves the effect of inert treatment by applying no thrust and strictly confining the manipulation to the skin surface [15]. SM accurately reflects the efficacy of FSM for RCRSP.

### Intervention description {11a}

Participants will receive FSM or SM in 4 therapy sessions once a week. Both groups will also receive the parallel daily home-based exercise program for 12 weeks.

### Five-step shoulder manipulation

1. Massage. The therapist stands behind the patient and kneads the sternoclavicular joint on the affected site with the index and middle fingers. The intensity is determined by the patient’s acid reflux sensation, and the pressure continued for 30 s.
2. Abduction. The therapist stands on the shoulder side of patient, assists the patient in abducting the affected shoulder to 90°, and applies appropriate upward and downward forces to allow the patient to engage in static confrontation with the relevant muscle groups resisting contraction for 30 s each time.
3. Release. The therapist stands behind the patient’s shoulder with one hand stabilizing the acromioclavicular joint, and the other hand hooking four fingers into the lower third of the medial edge of the scapula. The therapist passively abducts and adducts the scapula with both hands, reciprocates for 4–5 times.
4. Impact. The therapist stands behind the patient with the elbow flexed approximately 135° and the shoulder slightly prone and then holds the elbow in both hands and repeats transverse movements at an appropriate frequency to relax the shoulder. After complete shoulder relaxation, the therapist quickly lifts the humerus to the glenoid cavity to complete the internal impact of the glenohumeral joint.
5. Shake. The therapist stands on the side of the patient, crosses the right wrist with the patient’s forearm on the affected side, and then shakes the shoulder joint clockwise 3 times and then counterclockwise 3 times.

### Sham manipulation

The patient and therapist positioning will be the same as FSM, and the therapist will hold the position for several seconds, without performing the thrust. The operation is described as follows:

1. The therapist stands behind the patient and kneads the sternoclavicular joint on the affected site with the index and middle fingers. The operation is strictly confined to the surface level without involving the deep joint and lasts for 30 s.
2. The therapist stands on the side of the patient’s shoulder and assists the patient in abducting the affected shoulder to 90°. Maintain this position for 60 s without confrontation.
3. The therapist stands behind the patient’s shoulder with one hand on the acromioclavicular joint and the other hand on the lower third of the medial edge of the scapula. Kneading is strictly confined to the surface level and does not cause joint movement for 30 s.
4. The therapist stands behind the patient with around 135° elbow flexion and slight internal rotation of the shoulder and then holds the elbow with both hands and repeats transverse movements at an appropriation frequency.
5. Repeat step (2).

### Home-based exercise

The home-based exercise consists of 9 sections aimed at increasing the strength of the rotator cuff and scapular muscle. Some exercises require elastic theraband or hand weights to provide resistance, and the amount of resistance will vary depending on the individual. The protocol of the exercise program is as follows:

#### Weeks 1–6: exercises (1), (2), (3), (4), and (5)

1. Scapular setting. Seated isometric holding of the scapula in the extended and retracted position (5 repetitions, 5 s of each position)
2. Self-resisted isometric external rotation. Standing sideways to a wall, the elbow bent 45°, forearm pushing a towel roll into the wall with taking shoulder into external rotation (5 repetitions, 5 s of contraction each repetition)
3. Active external rotation. Sitting opposite a table, placing the forearm on the table, taking the shoulder into external rotation (2 sets, 10 repetitions)
4. Shoulder shrugs. Standing and elevating the scapula actively then lowering it slowly (2 sets, 10 repetitions)
5. Self-resisted isometric abduction. Standing sideways to a wall, the elbow bent 45° and pushing a towel roll into the wall with taking shoulder into the abduction (5 repetitions, 5 s of contraction each repetition)

#### Weeks 7–12: exercises (1), (6), (7), (8), and (9)

(6) Wall push up. Standing arm distance from the wall, shoulder flexion brings hands and shoulders level, body leaning forwards the wall with shoulders in 45° abduction and pushing away when reaching limit (2 sets, 5 repetitions)
(7) Resisted external rotation. Standing, elbow bent 90°, forearm close to abdomen, and grasping the theraband at navel level. Keeping upper arm close to the side of the body, and pulling the theraband with taking shoulder into external rotation (2 sets, 5 repetitions, using theraband)
(8) Resisted internal rotation. Standing, elbow bent 90°, forearm at 90° from abdomen, and grasping the theraband at navel level. Keeping upper arm close to the side of the body, and pulling the theraband with taking shoulder into internal rotation (2 sets, 5 repetitions, using theraband)
(9) Resisted abduction. Standing with arm down, holding a weight in hand, lifting hand with taking shoulder into abduction (2 sets, 10 repetitions, using the weight)

### Criteria for discontinuing or modifying allocated interventions {11b}

Participants may request to withdraw the study or be withdrawn because of study-related adverse events and withdrawal criteria. If a participant withdraws from the study, the study assistant will interview them about their reasons for leaving and collect their safety data, all of which will be recorded in detail in the case report forms (CRFs). If a participant experiences an adverse event and discontinues the study, the clinician will evaluate the patient’s condition to determine if additional treatment is needed.

### Strategies to improve adherence to interventions {11c}

During the study, registration and treatment will be free of charge. After each intervention, research assistants will help patients make an appointment for the next visit and remind patients of their appointment by phone or WeChat the day before the visit. In addition, an instructional video of home-based exercise, which can be viewed online, will be sent to the patients. Diary cards will also be provided, including the exact date and time of exercise, which patients will be asked to record daily and to present the completed cards to the research assistants for review at each visit.

### Relevant concomitant care permitted or prohibited during the trial {11d}

If unbearable pain occurs during the study, the use of non-steroidal anti-inflammatory drugs will be used on temporarily. The type of medication, dosage, and duration will be recorded in detail. If there is a conflict between non-steroidal anti-inflammatory drug use and follow-up, the follow-up will be delayed until 3 days after discontinuation of the medication.

### Provisions for post-trial care {30}

At the end of the study, patients in the SM group will receive four therapy sessions of FSM free of charge. All participants who complete the study will be able to continue to receive healthcare at their specific clinical trial settings, such as health education and functional exercise instruction. When patients complete the study but are not satisfied with symptom relief, clinicians will offer alternative treatments depending on the patient’s condition, such as medications, acupuncture, glucocorticoid injections, or surgery.

#### Outcomes {12}

Primary and secondary outcomes are presented in Table 1.

**Table 1:** Primary and secondary outcomes

	Primary outcome	Secondary outcomes		
Outcome measure	Shoulder function	Shoulder pain	Shoulder range of motion	Quality of life
Method of measurement	Constant-Murley Score [16]	Visual analog scale [17]	Active range of movement of shoulder joint [18]	The 36-Item Short Form Survey [19]
Analysis metric and method of aggregation	Difference in the mean of change from baseline between the two groups			
Time point	4, 12, 18, and 24 weeks. The principal analysis will be performed at 4 and 12 weeks	4, 12, 18, and 24 weeks		

### Primary outcome

#### CMS

The CMS, the most commonly used to assess pain and disability in patients with shoulder complaints [16], will be employed to measure the shoulder-specific scoring in the study. It consists of 100 points, including pain (15 points), activities of daily living (20 points), strength (25 points), and ROM: forward elevation, external rotation, abduction, and internal rotation of the shoulder (40 points); the higher the shoulder score, the better the shoulder function is [20]. The CMS has been confirmed to have good reproducibility (ICC = 0.827), reliability (Cronbach’s *α* = 0.739), and construct validity [16]. The research assistants assist the participants in completing the items and obtaining the final score.

### Secondary outcomes

#### VAS

The VAS score, a basic self-reported measure for assessing pain intensity, will be used in this investigation. It is a horizontal line of 100 mm, with 0 mm (the left endpoint) denoting no pain and 100 mm (the right endpoint) indicating excruciating pain [17]. The participants will be requested to choose the location on the line that best indicated their pain level. Furthermore, participants will be advised to measure the intensity of the primary pain for 4 times: overall pain intensity and pain intensity at rest, at night, and during movement.

#### ROM

ROM will be measured in degrees using a goniometer. The ROM measurement protocol will be based on a previous study [18] and include flexion–extension, isolated abduction, neutral external rotation, and internal rotation.

#### SF-36

The SF-36 will be used to evaluate the participants’ quality of life. This health survey questionnaire is a standardized, well-researched, self-reporting health measure scoring ranged from 0 (poor health) to 100 (good health), which includes 36 questions covering eight health domains [19].

### Participant timeline {13}

As shown as Table 2, participants in FSM and SM both groups are treated up to 4 weeks based on the interventions of each group, in parallel, participants in each group performed exercises at home for 12 weeks. For the follow-up time points, the participants will be assessed at baseline and 4, 12, 18, and 24 weeks.

**Table 2:**
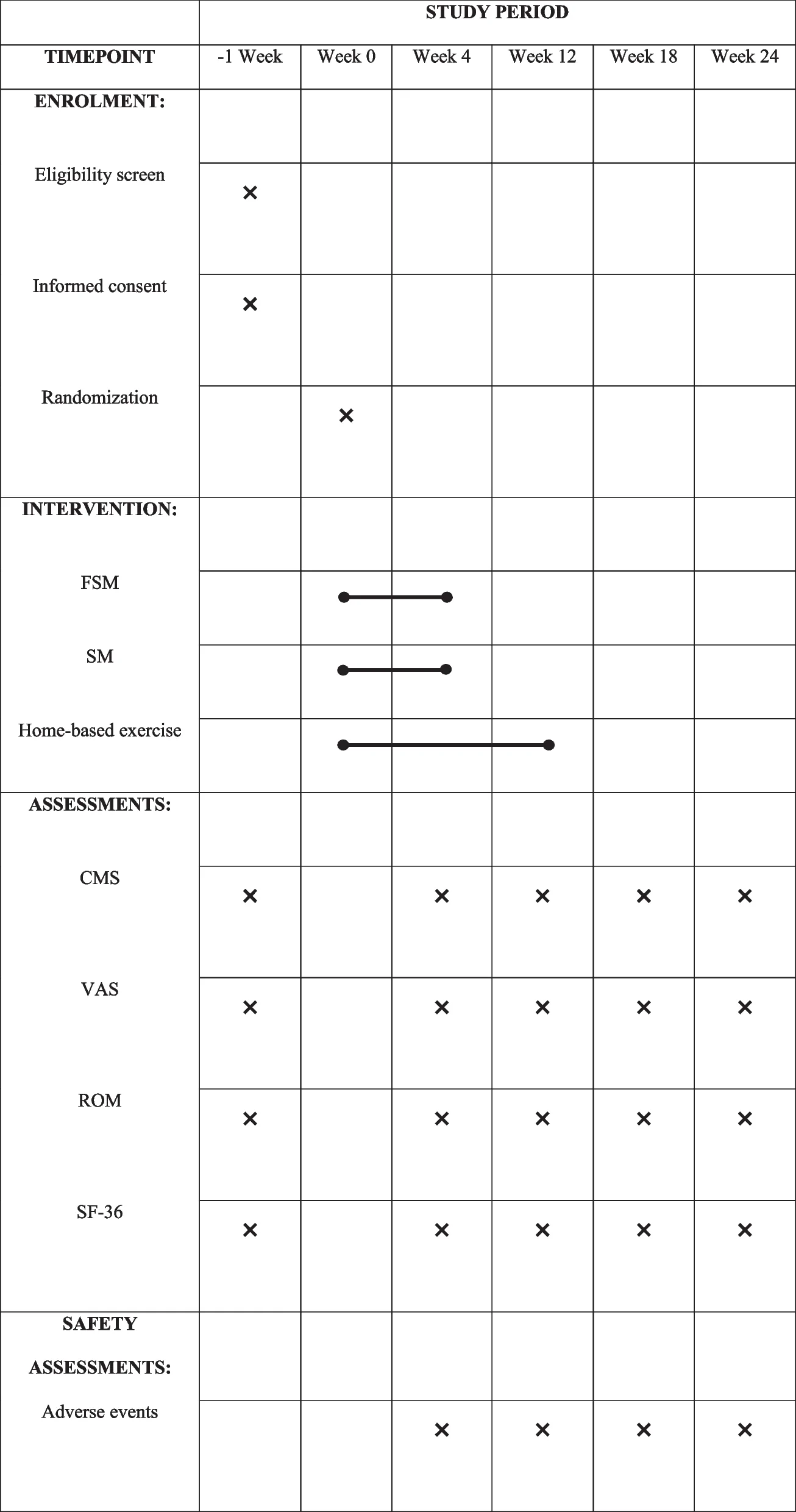
Schedule of enrolment, interventions, and assessment

*FSM* Five-step shoulder manipulation, *SM* Sham manipulation, *CMS* Constant-Murley score, *VAS* Visual analog scale, *ROM* Range of motion, *SF-36* 36-item short form survey

### Sample size {14}

The sample size is calculated based on our preliminary study, which was completed from November 2021 to February 2022. The prior research compared CMS changes from baseline to the end of 4-week treatment in patients receiving FSM or SM plus home-based exercise. Our previous results showed that CMS increased by 24.32 (μ_r_) in the FSM group and increased by 10.31 (μ_c_) in the SM group, and the standard deviation of the FSM group was 17.84 (*σ*). On the basis of the above data, when 1/3 of the standard deviation of the FSM group is selected for Δ, *α* = 0.025 (single-sided), *Z*_1−0.025_ = 1.96, 1 – *β* = 0.90, *Z*_1−0.10_ = 1.282, and the ratio of the number of subjects between the FSM group and SM group (*K*) = 1, using the following formula, 104 participants are required for each group: ${n}_{c}=\frac{{{\left({Z}_{1-\alpha }+{Z}_{1-\beta }\right)}^{2}\sigma }^{2}\left(1+\frac{1}{K}\right)}{{\left({\mu }_{r}-{\mu }_{c}-\Delta \right)}^{2}}$

$${n}_{r}={Kn }\ _{c}$$

On this basis, considering an estimated dropout rate of 20%, 125 cases will be needed for each group. Additionally, because there are three trial centers in this study, 15 additional cases are required for each group owing to the multicenter design. Therefore, a total of 280 participants will be enrolled in this study, with 140 participants in both the FSM group and the SM group.

### Recruitment {15}

Recruitment posters for the study will be posted at the three trial centers, and recruitment advertisements will be posted on the hospitals’ official WeChat accounts. Additionally, doctors at each trial center will inform patients who are assessed for shoulder pain about the study.

## Assignment of interventions: allocation

### Sequence generation {16a}

Eligible participants will be randomly assigned at a 1:1 ratio to either the FSM group or the SM group. The randomization scheme and allocation sequence will be generated via SAS PROC PLAN software (SAS Inc., Cary, NC, USA) by the Shanghai Medical Clinical Research Center (an independent third-party institution). The randomization list will be stratified by study center and will comprise the code, randomization number, and treatment regimen.

#### Concealment mechanism {16b}

The randomization list will be administered by the randomization manager who will not participate in other study procedures, and outcome assessors, data analysts-blinded and patients will be blinded to the randomization list.

#### Implementation {16c}

The randomization list will be generated by Shanghai Medical Clinical Research Center and submitted to the randomization manager for storage. Upon participant enrollment, the therapist will obtain the randomization number and treatment regimen for the patient via phone or WeChat, which will not be known by the patients and outcome assessors.

## Assignment of interventions: blinding

### Who will be blinded {17a}

Participants, outcome assessors, and data analysts will be blinded to the treatment allocation. To assess the effectiveness of the blinded approach, the participants and outcome assessors will answer a question at the week 12 evaluation point related to the allocation (“In your opinion, which intervention did you receive?”). The possible answers are as follows: (1) positive intervention; (2) sham intervention; or (3) I have no idea.

### Procedure for unblinding if needed {17b}

This study defines three unblinding situations: (1) when data entry is completed, (2) when statistical analysis is completed, and (3) when serious adverse events occur. All adverse events will be reported to the data and safety monitoring committee (DSMC), who will decide whether the patient’s participation needs to be discontinued and whether the patient should be unblinded immediately.

## Data collection and management

### Plans for assessment and collection of outcomes {18a}

Validated questionnaires and physical examinations were used to evaluate outcomes (Table 1).

The collection and recording of details of the chief complaint, demographics, clinical diagnosis, and details of medical and surgical history is the responsibility of the lead researchers.

Trained independent outcome assessors will be located at each study center. Before the start of the study, outcome assessors will receive standardized training to clarify their responsibilities and master the method of outcome evaluation. They will be responsible for collecting and recording symptoms using the methods listed in Table 1 and will be asked not to actively learn about patient groupings and interventions.

Patient-determined outcomes which include VAS [17], SF-36 scores [19], and CMS [16] will be self-reported by the patients, while ROM [18] and strength will require a standardized physical examination and recording by the research assistants. The effectiveness of the blinding will be assessed by research assistants who will ask questions and record the results at week 12. Adverse events, possible causes, whether the patient’s participation in the study is discontinued, and unblinding will also be recorded in detail by the research assistants.

### Plans to promote participant retention and complete follow-up {18b}

Follow-up visits (at weeks 18 and 24) will be scheduled by phone or WeChat. When the patients complete the follow-up, the transportation subsidy will be paid to promote participant retention and complete follow-up.

### Data management {19}

After the study is completed, the CRFs generated by each trial center will be submitted to the responsible unit of the study (Shanghai Municipal Hospital of TCM). Data entry will be performed separately by two independent research assistants and cross-checked to ensure the accuracy of the data. After data entry, the first unblinding will be performed, and the data will be divided into two groups based on the intervention. After ensuring that the details of the intervention are not displayed, the data will be given to the Statistical Center of Shanghai University of TCM for statistical analysis. When the statistical analysis is completed, the second unblinding will be performed, and the data will be locked. Only the senior supervisor will be authorized to open the data.

### Confidentiality {27}

Records generated during the study (e.g., informed consent and CRFs) will be kept in the responsible unit and locked at the end of the study, with only the senior supervisor having access to these files. The information will be used for this study only and will not be used for any other purpose.

### Plans for collection, laboratory evaluation, and storage of biological specimens for genetic or molecular analysis in this trial/future use {33}

N/A. No biological specimens will be involved in this study.

## Statistical methods

### Statistical methods for primary and secondary outcomes {20a}

SPSS V26 (IBM Corp., Armonk, NY, USA) will be used for both data input and analysis. Statistical analysis will be performed as follows. First, descriptive statistics will be used for all outcome measures at each measurement point to summarize the results. For normally and nonnormally distributed measurement data, the mean ± standard deviation, and median, will be used for the statistical description of the degree of trend, and dispersion, respectively. Second, differences in the mean change in primary and secondary outcomes from baseline to each time point between the groups will be compared using two independent samples *t*-tests or the Mann–Whitney *U*-test. Finally, chi-square tests will be used for blind evaluation. Statistical significance will be set at a *p*-value < 0.05 (two-sided). Table 3 summarizes the statistical analysis of primary and secondary outcomes.

**Table 3:** Variables, measures, and methods for statistical analysis of primary and secondary outcomes

Variable/outcome	Outcome measure	Methods of analysis
1) Primary		
The mean of change in shoulder function from baseline to 4, 12, 18, and 24 weeks	Constant-Murley score (continuous)	*T*-test or Mann–Whitney *U*-test^a^
2) Secondary		
The mean of change in shoulder pain from baseline to 4, 12, 18, and 24 weeks	Visual analogue scale (continuous)	*T*-test or Mann–Whitney *U*-test^a^
The mean of change in shoulder range of motion from baseline to 4, 12, 18, and 24 weeks	Active range of movement of shoulder joint (continuous)	*T*-test or Mann–Whitney *U*-test^a^
The mean of change in quality of life from baseline to 4, 12, 18, and 24 weeks	The 36-Item Short Form Survey (continuous)	*T*-test or Mann–Whitney *U*-test^a^

^a^For data conforming to normal distribution, *T*-test is used, and for data not conforming to normal distribution, Mann–Whitney *U*-test test is used

### Interim analyses {21b}

N/A. On the basis of previous observation and clinical practice experience, we judge that there will be no high-grade adverse events during the study; therefore, we have not set an interim analysis.

### Methods for additional analyses (e.g., subgroup analyses) {20b}

N/A. In this study, there are just two groups including FSM group and SM group, with no subgroups set up within the two groups. As a result, there is no additional analysis.

### Methods in analysis to handle protocol non-adherence and any statistical methods to handle missing data {20c}

All analyses will be based on the intention-to-treat principle. For instance, data for all participants will be analyzed in accordance with the randomized treatment assignment, even if there are cases of protocol non adherence. Since outpatient appointments are made for each treatment and follow-up, and participants can always contact the investigators for any advice they need, there will not be much missing data. When the case is dropped or the patient’s compliance is poor, the reasons for the drop or poor compliance must be detailed in the case observation form, and the patient’s understanding and support must be obtained by contacting the patient as frequently as possible. The case drop-off, patient compliance, and adverse reactions must be statistically described, compared, and analyzed among groups.

### Plans to give access to the full protocol, participant-level data, and statistical code {31c}

The protocol for this study is publicly available at China Registered Clinical Trial Registration Center (ChiCTR2000037577). At the end of the study, the data and analysis results will be available from the corresponding author upon reasonable request.

## Oversight and monitoring

### Composition of the coordinating center and trial steering committee {5d}

The trial steering committee, comprising the senior supervisor and the directors of each center, will be responsible for monitoring and coordinating the research and providing day-to-day support. The research group will conduct monthly inspections to follow the progress of the research and ensure that the trial process is standardized.

### Composition of the data monitoring committee, its role, and reporting structure {21a}

This study will designate a DSMC consisting of an orthopedic surgeon, ethicist, and a statistician and an assistant, who will be independent of the study. To ensure the quality and safety of the study, the DSMC will check the manipulation technique, CRFs, patient safety, data quality, and the descriptions of withdrawals or study dropouts every 2 weeks.

### Adverse event reporting and harms {22}

The safety of the participants will be monitored by the DSMC throughout the study. After each manipulation session and at each follow-up, the study assistant will ask the participants if there are any adverse events, such as worsening pain or progression of dysfunction, and record these events (if any) in detail. Adverse events will be reported to the DSMC, who will determine whether the participant needs to be discontinued and unblinded immediately.

### Frequency and plans for auditing trial conduct {23}

The Ethics Committee of Shanghai Municipal Hospital of TCM will conduct an audit of the study every 3 months to understand the progress, safety, and authenticity of the data and whether there are ethical violations. The members of the ethics committee are independent of the investigators and the sponsor.

### Plans for communicating important protocol amendments to relevant parties (e.g., trial participants, ethical committees) {25}

Any modification to the study protocol will be reported to the Ethics Committee of Shanghai Municipal Hospital of TCM for re-approval. If the modified content has any impact on the patients, the patients will be informed and requested to provide signed informed consent again. At the same time, registration changes will be synchronized.

### Dissemination plans {31a}

Whether the results are positive or negative, we will publish them in the peer-reviewed journal.

## Discussion

RCRSP is a growing worldwide health problem that causes shoulder pain and completely or partially stiffness. Increasing evidence has shown that dynamic constriction of the subacromial space with compression of the subacromial soft tissues is considered to be the key cause of chronic RCRSP [21–23]. Patients with RCRSP usually have tight shoulder muscles and joint capsule, which, in turn, may cause abnormal kinematics of the humerus, scapula, and clavicle, resulting in narrowing of the subacromial space and compression of the soft tissue through it. Therefore, the restoration of subacromial space stenosis is a therapeutic goal for RCRSP treatment. Anatomically, the activity of the shoulder joint includes the coordination of glenohumeral, scapothoracic jiont, sternoclavicular, and acromioclavicular four joints [24]. Previous studies have proven that manipulation therapy can effectively promote the rehabilitation of RCRSP, implying that manipulation is an alternative treatment for RCRSP [25, 26]. Despite the fact that the obvious therapeutic modalities predominantly include glenohumeral or scapulothoracic joint mobilization, spinal manipulation, and soft tissue release [26], we created the FSM to address four joints associated with shoulder motion, trying to quickly regain joint coordination and relieve subacromial soft tissue compression. We hypothesize that individuals using FSM will achieve faster improvements in symptoms and functional limitations compared with those who receive the sham manipulation. Therefore, a randomized, participant-, outcome assessor-, and data analyst-blinded, placebo-controlled study is required to confirm the efficacy of FSM in treating RCRSP.

In clinical trials, a placebo as a control treatment not only effectively evaluates the effectiveness of the treatment tested but also facilitates the implementation of blinding and eliminates several potential biases, such as patients’ dependence on therapists and expectations of treatment efficacy [27–29]. In manipulative studies, it is impossible to blind the therapist; therefore, using an inert manipulative therapy as a placebo to blind the participants is the best alternative. Previous studies have demonstrated the reliability of using SM as a placebo in manipulative studies [15, 30]. In this study, the inert treatment effect is achieved by applying no thrust and strictly confining the manipulation to the skin surface in accordance with the procedure tailored to FSM. In this study, the participants will be treated individually at different times to avoid communication and interference between participants. We hope to minimize potential differences by implementing the above interventions.

The main symptoms of RCRSP are pain, dysfunction, and loss of strength, and therefore a comprehensive evaluation tool is appropriate for the primary outcome. The evaluation content of the CMS basically covers all the symptoms of RCRSP and the adverse effects on quality of life, with good validity and responsiveness. Because the CMS is used widely in the assessment of shoulder diseases [31], we chose this measurement tool as the primary outcome of the study. Additionally, we will use VAS scores, ROM, and SF-36 scores to accurately quantify pain, function, and quality of life, respectively.

To provide a more accurate assessment, we intend to conduct this well-designed RCT to investigate both the efficacy and safety of RCRSP, contributing to worthy clinical evidence for manipulative therapy in RCRSP.

## Trial status

Recruitment is scheduled to begin on 1 July 2022, and the study will continue until 31 December 2023. The current protocol is version 2.0, June 19, 2022.

## Supplementary Information

**Additional file 1: Supplementary Table 1.** Trial registration data.

## Data Availability

At the end of the study, the data and analysis results will be available from the corresponding author (XFL) upon reasonable request.

## Abbreviations

RCRSP: Rotator cuff–related shoulder pain
FSM: Five-step shoulder manipulation
SM: Sham manipulation
CMS: Constant-Murley score
VAS: Visual analog scale
ROM: Range of motion
SF-36: 36-Item Short Form Survey
TCM: Traditional Chinese medicine
DSMC: Data and safety monitoring committee
CRF: Case report form
