# Correction: Efficacy of five-step shoulder manipulation for rotator cuff-related shoulder pain: protocol for a multicenter randomized controlled trial

**DOI:** 10.1186/s13063-026-10049-2

**Published:** 2026-10-18

**Authors:** Shuang Liu, Jin-Tao Liu, Lin Chen, Tian-You Fan, Xue-Jun Cui, Shao-Dan Cheng, Yan-Jiao Chen, Qi Shi, Chun-Chun Xue, Xiao-Feng Li

**Affiliations:** 1 Shanghai Municipal Hospital of Traditional Chinese Medicine, Shanghai University of Traditional Chinese Medicine, 274 Zhijiangzhong Road, Shanghai, 200071 China; 2 LongHua Hospital Shanghai University of Traditional Chinese Medicine, 725 Wanpingnan Road, Shanghai, 200032 China; 3 Suzhou Hospital of Traditional Chinese Medicine, 899 Wuzhongxi Road, Suzhou, Jiangsu 215009 China; 4 Shanghai GuangHua Hospital of Integrated Traditional Chinese and Western Medicine, 540 Xinhua Road, Shanghai, 200052 China; 5 Shanghai Research Institute of Acupuncture and Meridian, YueYang Hospital of Integrated Traditional Chinese and Western Medicine, 650 Wanpingnan Road, Shanghai, 200030 China; 6 Qi Shi’s Studio of Famous Chinese Medicine Physician, 274 Zhijiangzhong Road, Shanghai, 200032 China

**Correction: Trials 24, 498 (2023)**

**https://doi.org/10.1186/s13063-023-07540-5**

Following the publication of the original article [1], we were notified that the ethical approval number should be corrected from 2021SHL-KY-65-01 to 2021-SHL-KYYS-148.

The original article has been corrected.
