# Supplementary material for: Efficacy of five-step shoulder manipulation for rotator cuff-related shoulder pain: protocol for a multicenter randomized controlled trial
Source: Trials. 2023 Aug 7;24:498. doi: 10.1186/s13063-023-07540-5 (PMC10405406; doi:10.1186/s13063-023-07540-5)
Supplement: Supplementary file 1 — Additional file 1: Supplementary Table 1. Trial registration data. [file 13063_2023_7540_MOESM1_ESM.docx]

Supplementary Table 1 Trial registration data

| Data category | Information |
| --- | --- |
| Primary registry and trial identifying number | China Registered Clinical Trial Registration Center, ChiCTR2000037577. |
| Date of registration in primary registry | 29 August 2020 |
| Secondary identifying numbers | ChiMCTR2000003838 |
| Source(s) of monetary or material support | Medical Innovation Project of Shanghai Science and Technology Commission (No. 21Y11921300) |
| Primary sponsor | Shanghai Science and Technology Commission |
| Secondary sponsor(s) | Shanghai University of TCM, Shanghai Municipal Hospital of TCM |
| Contact for public queries | Xiaofeng Li, Ph.D. and M.D. (lixiaofeng0409@163.com) |
| Contact for scientific queries | Xiaofeng Li, Ph.D. and M.D. Shanghai Municipal Hospital of Traditional Chinese Medicine, Shanghai University of Traditional Chinese Medicine, Shanghai, China |
| Public title | Five-step shoulder manipulation for rotator cuff-related shoulder pain: a multi-center, randomized, controlled clinical study |
| Scientific title | Research on the curative effect of rotator cuff-related shoulder pain treated with five-step shoulder manipulation therapy |
| Countries of recruitment | China |
| Health condition(s) or problem(s) studied | Manipulation, rotator cuff-related shoulder pain |
|  | Active comparator: Five-step shoulder manipulation |
|  | Placebo comparator: Sham manipulation |
| Key inclusion and exclusion criteria | Inclusion criteria: Age 40-70 years old; pain in the anterolateral acromial area or pain with active shoulder elevation, with symptoms lasting for more than 3 months; have one positive sign in each of the following 4 categories: presence of pain when resisting humeral external rotation or abduction, Jobe Test, Hawkins Test, Neer Test; VAS score >= 40 mm. |
|  | Exclusion criteria: Clinical signs of massive rotator cuff tears (a tear of greater than 5 cm in diameter) or the presence of two or more tendon tears; glenohumeral osteoarthritis (OA) or other inflammatory arthritis; frozen shoulder; fracture or dislocation of shoulder; previous neck or shoulder surgery; osteoporosis; neurological disorders; tumor; pregnancy. |
| Study type | Interventional study |
|  | Allocation: randomized; Intervention model: parallel assignment; Masking: participant- and evaluator-blinded |
|  | Primary purpose: treatment |
| Date of first enrolment | August 2020 |
| Target sample size | 280 |
| Recruitment status | Recruiting |
| Primary outcome(s) | Difference in the mean of Constant-Murley Score changes from baseline between the two groups (time frame: 24 weeks; not designated as safety issue) |
| Key secondary outcomes | Difference in the mean of Visual Analogue Scale changes from baseline between the two groups (time frame: 24 weeks; not designated as safety issue); Difference in the mean of changes from baseline between the two groups (time frame: 24 weeks; not designated as safety issue); Difference in the mean of 36-Item Short Form Survey changes from baseline between the two groups (time frame: 24 weeks; not designated as safety issue) |
